# Optimization of Lithium–Sulfur Battery Performance via Nickel-Doped α-MnO_2_ Modified Separator

**DOI:** 10.3390/nano16080449

**Published:** 2026-04-09

**Authors:** Zhengtao Zhao, Lin Wan, Jiahui Chen, Huangqing Ye

**Affiliations:** 1 College of Chemistry, Chemical Engineering and Materials Science, Soochow University, Suzhou 215127, China; 2 College of Electronic Information, Hubei Engineering Institute, Huangshi 435003, China; 3 School of Materials and Energy, Foshan University, Foshan 528200, China; 4 College of Materials Science and Engineering, Shenzhen University, Shenzhen 518060, China

**Keywords:** lithium–sulfur batteries, catalyst, manganese dioxide, doping

## Abstract

Lithium–sulfur batteries (LSBs) offer a theoretical energy density of 2600 Wh kg^−1^ but suffer from the polysulfide shuttle effect, which causes rapid capacity decay and limits practical application. To address this, we developed a bifunctional separator coating using Ni-doped α-MnO_2_ combined with carbon nanotubes (Ni-MnO_2_/CNTs). Ni doping induces lattice expansion due to the larger Ni^2+^ ionic radius, modulating the electronic structure to create more active sites, enhance electrical conductivity, and improve polysulfide adsorption and redox kinetics. The needle-like morphology further strengthens physical/chemical confinement of polysulfides and accelerates conversion reactions. Batteries with the Ni-MnO_2_/CNTs-modified separator deliver a high-rate capacity of 813 mAh g^−1^ at 5 C and exhibit a low capacity decay rate of 0.0399% per cycle over 1500 cycles at 2 C. Even under high sulfur loading (∼10 mg cm^−2^) and lean electrolyte conditions (10 μL mg^−1^), the cell maintains stable cycling with a decay rate of 0.0929% per cycle over 300 cycles at 0.2 C. This lattice-modulation strategy on commercial separators provides a simple, effective pathway toward high-energy-density, long-life LSBs.

## 1. Introduction

### 1.1. Introduction to Lithium-Ion Batteries

Currently, the surge in energy demand and the accelerated depletion of fossil resources have made the development of clean energy and the promotion of a green energy transition an urgent necessity. However, most clean energy sources face challenges in storage and transportation [1]. In this context, lithium-ion batteries (LIBs) have emerged as a key energy storage technology due to their favorable portability, long-term stable cycling performance, and high safety [2]. After years of commercial development, LIBs have established a dominant position in the market with high technical maturity.

However, the development of LIBs is now facing a fundamental bottleneck: the performance of their electrode materials has gradually approached theoretical limits. Despite ongoing progress through engineering optimization, the inherently low theoretical energy density of LIBs [3,4,5,6] severely constrains further performance improvements. The current system struggles to meet the growing demand for high-energy-density energy storage devices. Therefore, the development of next-generation battery technology with breakthrough theoretical energy density is imperative.

### 1.2. Introduction to Lithium–Sulfur Batteries

Lithium–sulfur batteries (Li-S) demonstrate a theoretical energy density of up to 2600 Wh kg^−1^ [7] and a theoretical specific capacity of 1675 mAh g^−1^ [8] due to the multi-electron conversion reaction of the sulfur cathode and the high theoretical specific capacity of the lithium metal anode, making them a potential next-generation, high-energy-density energy storage system. However, their practical application is severely constrained by their short cycle life, primarily due to the following key challenges:

**Low electronic conductivity of the sulfur cathode** [9]: At room temperature, sulfur has extremely low conductivity (approximately 5 × 10^−30^ S cm^−1^), leading to difficulties in electron transport. This not only causes significant electrochemical polarization [10,11,12] but also forms a passivation layer at the electrode/electrolyte interface [13], hindering the rapid reaction kinetics of sulfur species, resulting in low active material utilization and rapid capacity decay.

**The shuttle effect of polysulfides** [14]: During discharge, long-chain polysulfides (Li_2_S_n_, 4 ≤ n ≤ 8) generated at the cathode dissolve into the electrolyte and diffuse through the separator to the lithium anode driven by concentration gradients [15,16,17,18]. At the anode surface, they are reduced to short-chain polysulfides, which then diffuse back to the cathode during charging and are oxidized. This continuous, reversible dissolution–diffusion reaction is referred to as the “shuttle effect.” It not only leads to an irreversible loss of active material [19,20,21] and reduced coulombic efficiency [22] but also severely corrodes the lithium anode, disrupting its interface stability, making it the core cause of deteriorating cycle performance.

**Significant volume expansion** [23]: When sulfur (S_8_) is converted into the discharge end-product lithium sulfide (Li_2_S), there is a significant change in density (~80% volume expansion). Repeated charge–discharge cycles subject the cathode structure to enormous mechanical stress, easily leading to structural damage in carbon/sulfur composite electrodes. Structural failure [24] not only reduces sulfur utilization but also accelerates polysulfide leakage through the resulting cracks, exacerbating the shuttle effect; simultaneously, the deposition of shuttle reaction byproducts further damages the electrode structure, creating a vicious cycle.

**Instability of the lithium anode and dendrite growth** [25]: Lithium metal anodes face issues of uneven lithium ion deposition during cycling, easily forming lithium dendrites. The high local electric field at the dendrite tips accelerates their longitudinal growth; once they pierce the separator, internal short circuits occur, posing severe safety risks. Additionally, the fracture of dendrites during cycling [26] forms “dead lithium” that is electrochemically inactive, causing permanent capacity loss while the continuously exposed fresh lithium surface continues to consume the electrolyte to form a thick and uneven solid electrolyte interphase (SEI) film, leading to increased interfacial impedance and kinetic imbalance, further deteriorating cycling performance [27].

Recently, lithium–sulfur batteries have also made significant breakthroughs in practical applications. For example, in 2025, the Hu Fangyuan team at the Dalian University of Technology conducted research in the field of wide-temperature-range polymer brush separators, developing polymer brush separators with intrinsic redox activity. These separators anchor polysulfides via polar groups and accelerate their conversion while regulating the uniform distribution of lithium-ion flux to suppress dendrite growth. This design simultaneously addresses high-temperature shuttle effects, low-temperature kinetic hysteresis, and dendrite issues. In the same year, a team from Beihang University and the Institute of High Energy Physics of the Chinese Academy of Sciences conducted research in the field of single-atom-catalyzed long-cycle batteries. They designed N-S co-doped porous carbon-anchored undercoordinated Ni-N_3_ single-atom catalysts (Ni-NSC), which enhanced polysulfide adsorption and conversion kinetics through symmetry-breaking charge transfer, improved sulfur redox reaction efficiency, and provided a technical pathway for long-life power batteries for electric vehicles, breaking through the cycle-life bottleneck of lithium–sulfur batteries. As such, lithium–sulfur battery technology is gradually entering the stage of large-scale industrialization, with its high-energy-density advantage poised to support the development of future high-efficiency energy storage systems.

### 1.3. How Lithium–Sulfur Batteries Work

Traditional lithium–sulfur batteries consist of a porous cathode made of sulfur-based composite materials [28], a high-theoretical-capacity metallic lithium foil [29] as the anode, and a functional separator [30] and electrolyte [31] system. The separator acts as an electronic insulating medium, enabling selective lithium ion transmission through its microporous structure while inhibiting the migration of polysulfides; the lithium anode uses commercial-grade lithium foil, leveraging its low redox potential and high specific capacity to provide charge carriers [32]. The electrolyte is typically an ether-based electrolyte containing lithium salts, with lithium bis (trifluoromethane) sulfonyl imide (LiTFSI) being the most commonly used, which exhibits high ionic conductivity. The charging and discharging process of lithium–sulfur batteries is relatively complex, involving a solid–liquid–solid phase transition process [33].

The overall equation for the sulfur reduction reaction (SRR) during discharge in lithium–sulfur batteries is:

S_8_ + 16Li → 8Li_2_S (1)

The specific reaction is primarily divided into the following four stages:

Stage I: S_8_ (s) → Li_2_S_8_ (l) (2)

Stage II: Li_2_S_8_ (l) → Li_2_S_6_ (l) → Li_2_S_4_ (l) (3)

Stage III: Li_2_S_4_ (l) → Li_2_S_2_ (s)/Li_2_S (s) (4)

Stage IV: Li_2_S_2_ (s) → Li_2_S (s) (5)

### 1.4. Research Motivation and Scope

In this work, MnO_2_ is regarded as a catalytic material that can promote the redox reactions of sulfur-containing species in lithium–sulfur batteries. It is mainly introduced as a functional layer on the separator, which is located between the cathode and anode. This configuration helps to facilitate the conversion of polysulfides and reduce their shuttle behavior. Despite the promise of transition metal compounds like MnO_2_ as catalytic materials for lithium–sulfur batteries, their practical application is hindered by insufficient active sites, poor conductivity, and weak polysulfide adsorption, which limit their ability to suppress the shuttle effect and enhance redox kinetics. To address these challenges, this study proposes a lattice-modulation strategy via nickel (Ni) doping into α-MnO_2_ to construct a bifunctional separator coating. The ionic radius mismatch induces lattice expansion and electronic structure modulation, creating additional active sites and improving charge transfer. Integrated with CNTs, the Ni-MnO_2_ catalyst demonstrates strongly enhanced polysulfide adsorption and conversion kinetics, achieving good rate performance and long-term cycling stability even under high sulfur loading and lean electrolyte conditions, offering a potential strategy for practical high-energy-density lithium–sulfur batteries.

## 2. Experimental Section

### 2.1. Material Synthesis and Battery Assembly

#### 2.1.1. Cathode Preparation

**Conventional lithium–sulfur battery cathode**: Ensaco 350G and sulfur powder was thoroughly ground at a mass ratio of 1:4, then heated at 155 °C for 12 h in an argon atmosphere. The resulting carbon–sulfur composite material was thoroughly ground with Super C65 (one kind of conductive carbon black, mainly composed of elemental carbon) and PVDF at a mass ratio of 8:1:1 [34], and an appropriate amount of NMP was added to form a uniform slurry. The slurry was then coated onto the surface of carbon paper and vacuum-dried at 60 °C for 8 h. The resulting composite was then cut into circular disks with a diameter of 12 mm using a slicing machine, with the sulfur loading area of each disk controlled to approximately 1.5 mg cm^−2^. Ensaco 350G, supplied by Imerys Graphite and Carbon (Bodio, Switzerland), is a conductive carbon black (mainly composed of elemental carbon) with good electrical conductivity and structural stability. It shows better conductivity and structural stability than Ensaco 240 and other common carbon blacks and is used as the conductive matrix in the sulfur cathode.

**High-sulfur-loaded cathode**: Ensaco 350G was thoroughly ground with sulfur powder at a mass ratio of 1:5, and the mixture was heated at 155 °C for 12 h in an argon atmosphere, followed by heating at 300 °C for 1 h. The resulting carbon–sulfur composite material was thoroughly ground with LA136DL at a mass ratio of 9:1, and an appropriate amount of deionized water and ethanol was added to form a uniform slurry. The slurry was then poured onto the surface of carbon paper and vacuum-dried at 60 °C for 8 h. Finally, it was cut into circular disks with a diameter of 12 mm using a slicing machine. During high sulfur loading tests, the areal loading of sulfur on the cathode was 7.0–9.0 mg cm^−2^. LA136DL is a water-based acrylic binder for lithium–sulfur battery electrodes, purchased from Shenzhen Kejing Star Technology Co., Ltd. (Kejing, Shenzhen, China).

#### 2.1.2. Modified Separator Preparation

Synthesis of Ni-MnO_2_: Initially, potassium permanganate (KMnO_4_, 1.185 g, 7.5 mmol) and manganese sulfate monohydrate (MnSO_4_·H_2_O, 0.5 g, 3.27 mmol) were dissolved in deionized water (120 mL). The solution was stirred vigorously for 60 min. Subsequently, nickel chloride hexahydrate (NiCl_2_·6H_2_O, 0.4 g, 1.68 mmol) was introduced into the above mixture, followed by another hour of stirring. The resultant solution was then transferred into a Teflon-lined stainless-steel autoclave and subjected to hydrothermal treatment at 180 °C for 16 h. After cooling to room temperature naturally, the precipitate was collected by filtration, washed thoroughly with deionized water and ethanol via ultrasonication (three cycles), and finally dried to obtain the Ni-MnO_2_ material.

Fabrication of Modified Separator: The Ni-MnO_2_ material, carbon nanotubes (CNTs), and polyvinylidene fluoride (PVDF) were mixed in a mass ratio of 6:3:1. This mixture was dispersed in ethanol (240 mL) and ultrasonicated for 2 h to form a homogeneous slurry. A controlled amount of the resulting slurry was then uniformly coated onto a conventional Celgard 2325 separator via vacuum filtration [32]. The coated separator was dried at 60 °C for 12 h in a vacuum oven to yield the final Ni-MnO_2_/CNTs-modified separator. Celgard 2325 (Celgard, LLC, Charlotte, NC, USA) is a PP/PE/PP triple-layer microporous separator with favorable thermal stability.

Conventional battery: The catalyst material, Super C65 (Imerys Graphite & Carbon, Bodio, Switzerland), and PVDF (Sigma-Aldrich, St. Louis, MO, USA) were dispersed in ethanol at a mass ratio of 5:4:1, sonicated for 30 min, and then filtered onto the surface of the Celgard 2325 separator [35].

High-sulfur-poor-electrolyte battery: The catalyst material, Super C65, and PVDF were dispersed in NMP (Shanghai Macklin Biochemical Co., Ltd., Shanghai, China) at a mass ratio of 3:6:1, stirred into a uniform slurry [36], and coated onto the surface of the Celgard 2325 separator.

#### 2.1.3. Preparation of Li_2_S_6_ and Li_2_S_8_ Solutions

In an argon-filled glove box (Mikrouna Mech-Electronic Equipment Co., Ltd., Shanghai, China), weigh 183.81 mg of lithium sulfide (Li_2_S) and 641.40 mg of sulfur powder, dissolve in 10 mL of DOL/DME solvent, and stir vigorously for 3 days to obtain a 0.2 M Li_2_S_6_ solution. Stir thoroughly until clear before each use. The preparation method for the 0.2 M Li_2_S_8_ solution is the same as above, with 897.70 mg of sulfur powder added. DOL = 1,3-dioxolane, and DME = 1,2-dimethoxyethane.

#### 2.1.4. Battery Assembly

Battery assembly was performed in a glove box, with O_2_ and H_2_O levels both below 0.1 ppm. The battery was assembled in the following order: positive electrode casing, positive electrode plate, electrolyte, separator, electrolyte, lithium plate, spacer, spring, and negative electrode casing. The battery casing used was a CR 2032 battery casing (304 stainless steel, with springs and spacers, Hefei Kejing Materials Technology Co., Ltd., Hefei, China). The electrolyte–sulfur ratio for conventional batteries is controlled around 40 µL mg^−1^. For high-sulfur, lean-electrolyte batteries, the electrolyte–sulfur ratio was controlled at approximately 10 μL mg^−1^. The electrolyte uses a mixture of DOL and DME as solvents in a 1:1 volume ratio and also contains 1 M LiTFSI and 2% wt LiNO_3_ as electrolyte additives. CV was performed at a scan rate of 0.1 mV/s, and EIS was measured over a frequency range from 0.1 Hz to 100,000 Hz. All electrochemical measurements were performed on at least three independent batteries to ensure good reproducibility, and representative results are presented in this work.

### 2.2. Characterization

Scanning electron microscopy (SEM) images were recorded on a field emission scanning electron microscope (SEM, Nova NanoSEM 450, FEI Company, Hillsboro, OR, USA). The X-ray diffraction (XRD, Rigaku D/Max 2500, Rigaku Corporation, Tokyo, Japan) with Cu-Kα radiation was used for measurement of the products. Ultraviolet-visible spectra of different catalysts after immersion in Li_2_S_6_ solution were characterized by UV-Vis instrument Lambda 35 UV-Vis spectrophotometer (Perkin-Elmer Inst., Norwalk, CT, USA).

## 3. Results and Discussion

### 3.1. SEM and XRD of Material

**Figure 1:**
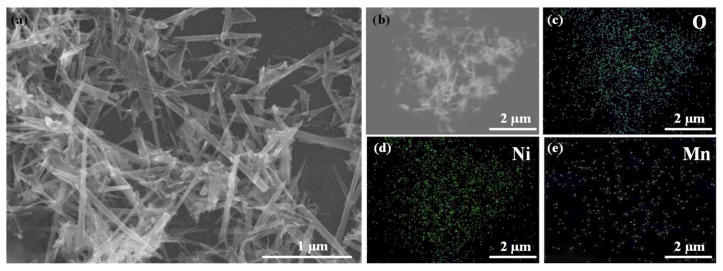
(**a**,**b**) Show SEM images of Ni-MnO_2_ material, and (**c**–**e**) show EDX mapping.

This study investigates the morphology and microstructure of the catalyst using scanning electron microscopy (SEM). As shown in Figure 1, the synthesized Ni-MnO_2_ exhibits a needle-like microstructure [37]. The needle-like morphology with moderate curvature provides a large surface area and abundant accessible active sites, which is favorable for charge transfer and polysulfide adsorption/conversion, as supported by previous reports on nanostructured electrocatalysts for Li–S batteries [38]. The EDX elemental mappings confirm the coexistence of Mn, O, and Ni elements in the as-prepared Ni-MnO_2_ sample, demonstrating the successful introduction of Ni species into the material. Combined with the crystal structure characteristics of α-MnO_2_, this result suggests that nickel ions enter the [MnO_6_] octahedral framework through lattice substitution, occupying part of the Mn sites, thereby exposing more active sites on the catalyst [39].

**Figure 2:**
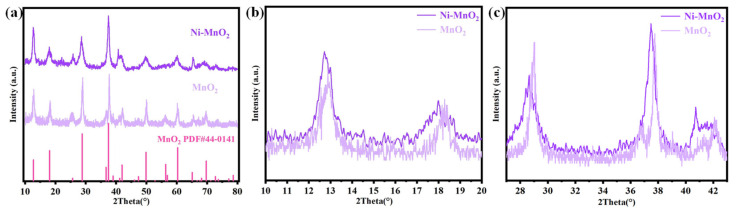
(**a**) XRD patterns of Ni-MnO_2_ and MnO_2_ and (**b**,**c**) magnified views of the XRD patterns.

Further XRD phase analysis was conducted on the products of the Ni-MnO_2_ catalyst synthesis process. Figure 2 shows the X-ray diffraction (XRD) patterns of the Ni-MnO_2_ composite catalyst and its undoped control sample. By comparing with the standard PDF card for manganese dioxide (PDF#44-0141), it was found that the Ni-doped MnO_2_ catalyst material exhibits distinct characteristic diffraction peaks at 2θ = 12.8°, 18.1°, 28.9°, 37.6°, 41.8°, 49.9°, 56.4°, and 60.3°, corresponding to the (110), (200), (310), (211), (301), (411), (600), (521) crystal planes of MnO_2_, respectively. The diffraction peaks exhibit narrow half-widths and sharp peak shapes, indicating that the material has a high degree of crystallinity [40]. Notably, no new impurity peaks were observed in the doped material, suggesting that the introduction of Ni does not alter the basic crystal structure of the α phase of MnO_2_ and does not generate other byproducts. To further investigate the effect of doping on the microstructure of the MnO_2_ catalyst, Figure 2b,c jointly present the magnified spectra of the (110), (200), (310), and (211) characteristic peaks. By comparing the XRD peaks of Ni-MnO_2_ and MnO_2_ samples, it is clearly observed that the diffraction peaks of the Ni-MnO_2_ sample exhibit a leftward shift of approximately 0.15° compared to pure MnO_2_. This is probably because when Ni atoms replace part of the Mn and are doped into the MnO_2_ crystal lattice, the larger radius of Ni compared to Mn causes lattice expansion when Ni^2+^ occupies Mn sites, leading to an increase in interplanar spacing and a corresponding decrease in the diffraction angle θ.

### 3.2. Adsorption Performance Test

**Figure 3:**
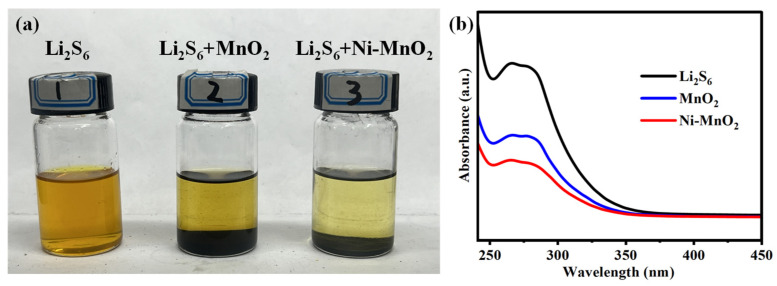
(**a**) Visual adsorption experiment images and (**b**) ultraviolet-visible spectra of different catalysts after immersion in Li_2_S_6_ solution.

To investigate the adsorption capacity of the prepared catalyst for polysulfides, equal masses of Ni-MnO_2_ and MnO_2_ powders were separately immersed in Li_2_S_6_/DOL-DME solutions and left to stand for 12 h in an argon glove box for visual adsorption experiments. As shown in Figure 3a, the solution containing the Ni-MnO_2_ catalyst exhibited a colorless and transparent state, while the MnO_2_ group solution remained distinctly pale yellow, indicating that the upper layer of the MnO_2_ group solution still contained a noticeable amount of polysulfides. The above experimental comparison demonstrates that Ni doping enhances the material’s chemical anchoring effect on soluble polysulfides [41]. To further quantify the adsorption strength of the material toward polysulfides, UV-Vis spectroscopy analysis was performed on the upper clear solution after adsorption (Figure 3b). The Li_2_S_6_ solution without added catalyst exhibited a typical polysulfide S_6_^2−^ characteristic absorption peak at 280 nm, while the Li_2_S_6_ solution with the Ni-MnO_2_ catalyst exhibited the lowest absorbance in the aforementioned wavelength range. According to the Lambert–Beer law [42], this indicates the lowest polysulfide content in the supernatant, confirming that the catalyst possesses the strongest polysulfide adsorption capacity. This adsorption behavior not only suppresses the dissolution and diffusion of polysulfides but also promotes solid–liquid conversion kinetics by reducing the nucleation overpotential of Li_2_S, thereby establishing an adsorption–conversion synergy mechanism to enhance the cycling stability of lithium–sulfur batteries.

### 3.3. Electrochemical Results

**Figure 4:**
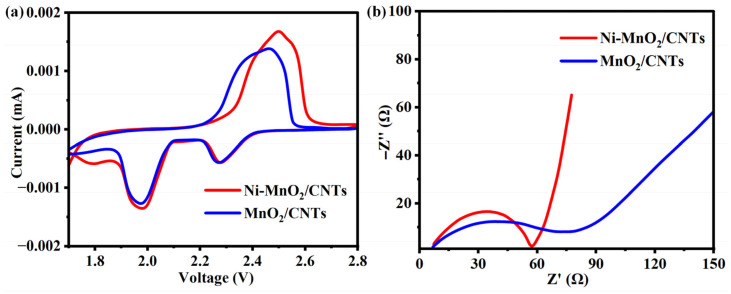
(**a**) CV curves and (**b**) EIS curves of Ni-MnO_2_/CNTs and MnO_2_/CNTs.

To investigate the kinetics of redox reactions, we modified the separator with two materials, Ni-MnO_2_/CNTs and MnO_2_/CNTs, and assembled lithium–sulfur batteries for electrochemical impedance testing and cyclic voltammetry testing. As shown in Figure 4a, the battery with the Ni-MnO_2_/CNTs separator exhibited a higher peak current value, indicating stronger conversion kinetics and faster reaction rates [11]. It is noteworthy that the CV peaks for the Ni-MnO_2_/CNTs cell appear at slightly higher overpotentials compared to the MnO_2_/CNTs cell. The slightly higher overpotential originates from the stronger chemical adsorption of polysulfides on Ni-MnO_2_/CNTs, which moderately increases the initial activation energy for initiating the conversion reaction. In contrast, the higher peak current in CV and smaller charge-transfer resistance (Rct) in EIS consistently demonstrate faster interfacial charge transfer and higher overall reaction dynamics after activation, owing to the improved electronic conductivity by Ni doping. The strong adsorption helps to suppress polysulfide shuttling, while the enhanced charge transfer ensures rapid redox kinetics; these two factors work synergistically to contribute to the enhanced electrochemical performance, rather than being contradictory [43]. The overall enhancement in reaction kinetics outweighs the minor increase in activation overpotential. As shown in Figure 4b, the battery with the Ni-MnO_2_/CNTs separator has a smaller semicircle in the high-frequency region, indicating a lower charge-transfer resistance, while the slope of the tail in the low-frequency region is steeper, suggesting a higher charge-transfer rate of the surface Ni-MnO_2_/CNTs. This indicates faster charge-transfer and lithium-ion diffusion rates, further confirming the enhanced redox kinetics [44]. The apparent discrepancy between the lower charge-transfer resistance (Rct) observed in EIS and the slightly higher overpotential in CV can be reconciled by considering the different states of the battery during these measurements. EIS was measured at the open-circuit potential (a steady-state, non-destructive measurement), primarily probing the intrinsic conductivity and charge transfer capability of the electrode/electrolyte interface. The lower Rct for Ni-MnO_2_/CNTs indicates its enhanced inherent conductivity and more efficient charge transfer interface. In contrast, CV is a dynamic technique performed over a wide potential range. The slightly higher overpotential suggests that the energy barrier for activating the conversion of polysulfides (the activation overpotential) might be marginally higher for Ni-MnO_2_/CNTs, likely due to its stronger polysulfide adsorption, as discussed earlier. However, once the reaction is activated, the lower Rct ensures faster kinetics throughout the process, leading to higher peak currents and better overall performance. Thus, the two techniques provide complementary information about different aspects of the electrochemical process.

**Figure 5:**
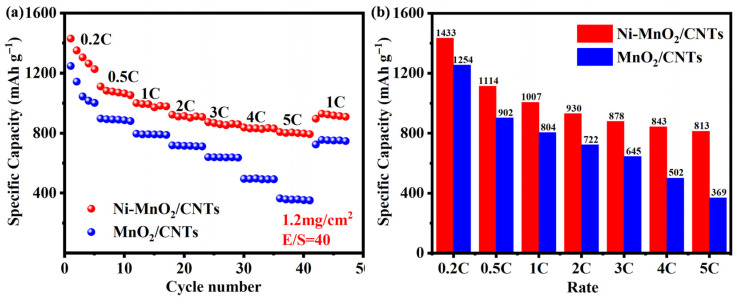
(**a**) Rate curves and (**b**) specific discharge capacity at different rates for Ni-MnO_2_/CNTs and MnO_2_/CNTs.

The battery rate performance curve is an important tool for evaluating a battery’s ability to charge and discharge rapidly. As shown in Figure 5a, under conditions of 1.2 mg cm^−2^ sulfur loading and a liquid sulfur ratio of 40 μL mg^−1^, the rate performance of the Ni-MnO_2_/CNTs separator battery was improved. It delivers specific capacities of 1433.4, 1114.0, 1007.4, 929.6, 878.1, 843.2, and 813.4 mAh g^−1^ at rates of 0.2 C, 0.5 C, 1 C, 2 C, 3 C, 4 C, and 5 C, respectively. After the current density was reduced back to 1 C, the discharge capacity showed good recovery, confirming high reversibility. In contrast, batteries prepared using MnO_2_/CNTs exhibited lower specific capacity at all tested current densities, with a specific capacity of 368.8 mAh g^−1^ at 5 C (45% of that of the Ni-MnO_2_/CNTs separator battery). Compared to MnO_2_/CNTs, Ni-MnO_2_/CNTs exhibit higher capacity and better rate performance, indicating that the Ni-doped battery demonstrates enhanced electronic and ionic conductivity, improving reaction kinetics. Figure 5b shows a numerical comparison of rate performance, with the bar chart clearly illustrating that the Ni-MnO_2_/CNTs-modified separator exhibits a higher specific capacity than the MnO_2_/CNTs separator at the same rate.

**Figure 6:**
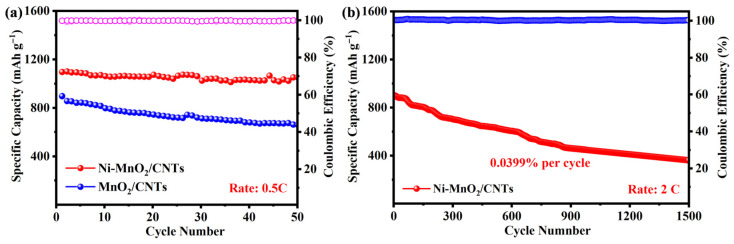
(**a**) Short-cycle curves of Ni-MnO_2_/CNTs and MnO_2_/CNTs at 0.5 C and (**b**) long-cycle curves of Ni-MnO_2_/CNTs at 2 C. The Coulombic efficiencies for Ni-MnO_2_/CNTs at 0.5 C and 2 C are indicated by hollow pink and blue dots in (**a**) and (**b**), respectively.

Battery capacity degradation is a key indicator of battery lifespan. As shown in Figure 6a, under a current density of 0.5 C, after 50 cycles of testing, the Ni-MnO_2_/CNTs separator battery exhibits higher specific capacity, lower capacity degradation rate, and longer lifespan. As shown in Figure 6b, under a current density of 2 C, after 1500 cycles of testing, the average capacity decay rate of the Ni-MnO_2_/CNTs separator battery was 0.0399% per cycle. The battery’s lifespan was extended compared to separators doped with Ni atoms. Although the Ni-MnO_2_/CNTs cell demonstrates a low average decay rate of 0.0399% per cycle, the gradual capacity fade over 1500 cycles is inevitable and can be attributed to several factors beyond the catalyst’s capability. Firstly, even with the effective suppression of the shuttle effect by the modified separator, the gradual consumption of the electrolyte and lithium anode due to side reactions (e.g., continuous breakdown and reformation of the SEI layer on lithium metal) remains a fundamental challenge in Li–S batteries. Secondly, the irreversible loss of active sulfur species trapped in the cathode structure or within the separator layer, which cannot be fully re-utilized over prolonged cycling, also contributes to the capacity degradation. Thirdly, the possible mechanical degradation or partial detachment of the catalytic coating on the separator during long-term cycling might slightly reduce its efficacy. Addressing the lithium anode degradation through advanced anode protection strategies would be crucial for further improving the long-term capacity retention.

**Figure 7:**
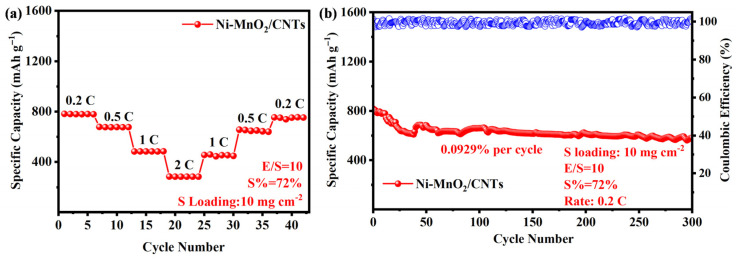
High sulfur loading (**a**) rate performance under poor electrolyte conditions, (**b**) cycling performance at a current density of 0.2 C. The Coulombic efficiency for Ni-MnO_2_/CNTs at 0.2 C is shown as hollow blue dots in (**b**).

In actual production and use, batteries often need to achieve higher energy density under conditions of low electrolyte amount and high sulfur loading. Therefore, we conducted further tests on the rate performance and cycling performance of Ni-MnO_2_/CNTs separator batteries under conditions of 10 mg cm^−2^ high sulfur loading and 10 μL mg^−1^ low liquid–sulfur ratio. As shown in Figure 7a, when the current density first increases and then decreases, the rate performance curve exhibits a symmetrical trend, indicating good reversibility of the battery. As shown in Figure 7b, under a current density of 0.2 C, after 300 cycles of testing, the average decay rate of the Ni-MnO_2_/CNTs separator battery was 0.0929% per cycle. These results indicate that the Ni-MnO_2_/CNTs separator battery has good practical application potential.

## 4. Conclusions

This study successfully constructed a Ni/MnO_2_ bifunctional catalyst with a high-curvature needle-like structure through a nickel ion lattice doping strategy. Ni substitution at Mn sites induces lattice expansion, and the resulting unique micro-nano structure enhances polysulfide adsorption through localized electric field concentration effects. Concurrent symmetry breaking optimizes the electronic structure, and the exposed Ni-Mn synergistic active sites accelerate liquid–solid conversion kinetics, reducing charge-transfer impedance by 40% and increasing redox reaction rates by 1.8 times. Batteries based on Ni-MnO_2_/CNTs-modified separators achieve a reversible capacity of 813 mAh g^−1^ at a high rate of 5 C (a 120% increase over the undoped system) and exhibit a low decay rate (only 0.0399% per cycle after 1500 cycles). Under harsh conditions of 10 mg cm^−2^ high sulfur loading and low electrolyte amount (10 μL mg^−1^), the battery maintains stable cycling performance of 0.0929% per cycle over 300 cycles, highlighting its potential for industrial application. This strategy provides a design approach for modifying separators in high-energy-density lithium–sulfur batteries.

## Data Availability

The original contributions presented in this study are included in the article. Further inquiries can be directed to the corresponding authors.
